# The frequency of *kdr* and *ace-1* alleles in *Anopheles gambiae* s.l. before and during indoor residual spraying (IRS) implementation and four years after IRS withdrawal in three districts in Atacora, Benin

**DOI:** 10.1186/s13071-024-06206-8

**Published:** 2024-03-07

**Authors:** Esdras Mahoutin Odjo, Daniel Impoinvil, Arsène Jacques Y. H. Fassinou, Gil Germain Padonou, Rock Aïkpon, Albert Sourou Salako, André Aimé Sominahouin, Constantin Adoha, Boulais Yovogan, Razaki Osse, Olivier Oussou, Filémon Tokponnon, Virgile Gnanguénon, Ahmed Saadani Hassani, Martin C. Akogbeto

**Affiliations:** 1 Centre de Recherche Entomologique de Cotonou, Cotonou, Bénin; 2 Faculté des Sciences et Techniques—Université d’Abomey-Calavi, Abomey Calavi, Bénin; 3 U.S. President’s Malaria Initiative (PMI), U.S. Centers for Disease Control and Prevention, Atlanta, GA USA; 4 Ecole Normale Supérieure de Natitingou, Université Nationale des Sciences, Technologies, Ingénierie et Mathématiques (UNSTIM) d’Abomey, Abomey, Bénin; 5 Université Nationale d’Agriculture de Porto-Novo, Porto-Novo, Bénin; 6 US President’s Malaria Initiative (PMI), U.S. Agency for International Development (USAID), Cotonou, Benin; 7 US President’s Malaria Initiative (PMI), U.S. Centers for Disease Control and Prevention (CDC), Cotonou, Benin

**Keywords:** *Anopheles gambiae*, IRS withdrawal, Genetic structure, Resistance, Benin

## Abstract

**Background:**

Indoor residual spraying (IRS) was first implemented in the Atacora department, Benin from 2011 to 2012 using bendiocarb (carbamate) followed by annual spraying with pirimiphos-methyl (organophosphate) from 2013 to 2018. Before and after IRS implementation in Atacora, standard pyrethroid insecticide-treated bed nets were the main method of vector control in the area. This study investigated the knockdown resistance (*kdr*) gene (*L1014F)* and the acetylcholinesterase (*ace-1*) gene (*G119S*), before and during IRS implementation, and 4-years after IRS withdrawal from Atacora. This was done to assess how changes in insecticide pressure from indoor residual spraying may have altered the genotypic resistance profile of *Anopheles gambiae* s.l.

**Method:**

Identification of sibling species of *An. gambiae* s.l. and detection of the *L1014F* mutation in the *kdr* gene and *G119S* mutation in *ace-1* genes was done using molecular analysis. Allelic and genotypic frequencies were calculated and compared with each other before and during IRS implementation and 4 years after IRS withdrawal. The Hardy–Weinberg equilibrium and genetic differentiation within and between populations were assessed.

**Results:**

Prevalence of the *L1014F* mutation in all geographic *An. gambiae* s.l. (*An*. *gambiae* s.s., *Anopheles*. *coluzzii*, *Anopheles*. *arabiensis*, and hybrids of “*An*. *gambiae* s.s. and *An*. *coluzzii*”) populations increased from 69% before IRS to 87% and 90% during and after IRS. The *G119S* allele frequency during IRS (20%) was significantly higher than before IRS implementation (2%). Four years after IRS withdrawal, allele frequencies returned to similar levels as before IRS (3%). Four years after IRS withdrawal, the populations showed excess heterozygosity at the *ace-1* gene and deficit heterozygosity at the *kdr* gene, whereas both genes had excess heterozygosity before and during IRS (*F*_*IS*_ < 0). No genetic differentiation was observed within the populations.

**Conclusions:**

This study shows that the withdrawal of IRS with bendiocarb and pirimiphos-methyl may have slowed down the selection of individual mosquitoes with *ace-1* resistance alleles in contrast to populations of *An*. *gambiae* s.l. with the *L1014F* resistance allele of the *kdr* gene. This may suggest that withdrawing the use of carbamates or organophosphates from IRS or rotating alternative insecticides with different modes of action may slow the development of *ace-1* insecticide-resistance mutations. The increase in the prevalence of the *L1014F* mutation of the *kdr* gene in the population, despite the cessation of IRS, could be explained by the growing use of pyrethroids and DDT in agriculture and for other domestic use. More observational studies in countries where carbamates or organophosphates are still being used as public health insecticides may provide additional insights into these associations.

**Graphical Abstract:**

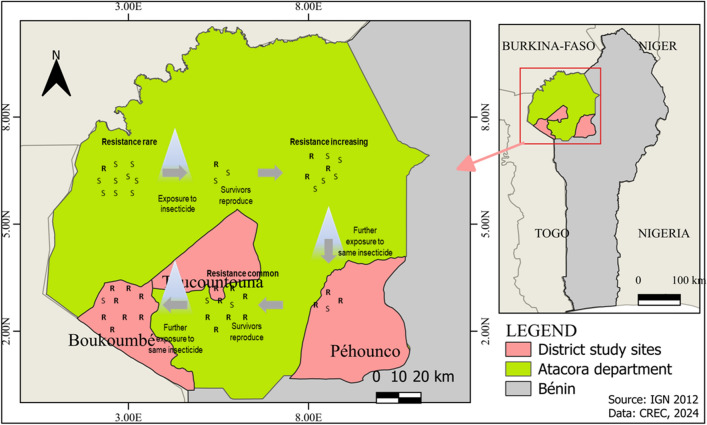

## Background

The massive use of insecticide-based vector control interventions such as indoor residual spraying (IRS) contributes to the insecticide resistance of target vectors [1, 2]. In Africa, and particularly in Benin, high insecticide resistance has been documented in various vector populations in the different intervention areas [3–6]. Various insecticide detoxification mechanisms have been described, and some are likely mediated through the modification of the genetic structure [2, 7–10]. For example, mutations in sodium channel modulators/knockdown resistance gene of mosquitoes are thought to induce insecticide resistance to pyrethroids, while mutations in the acetylcholinesterase gene (*ace*-1) can induce resistance to carbamates or organophosphates [11].

In Benin, large-scale (blanket spray: geographic-wide) IRS with Ficam M^®^ (bendiocarb 800 *g*/kg; carbamate) occurred from 2008 to 2010 in the southern Benin districts of Adjohoun, Akpro-Misserete, Dangbo, and Semé-Podji located in the Ouémé department [12, 13]. Benin shifted the IRS program to the Atacora district in the northern of the country, where Ficam M^®^ was used in 2011 and in 2012 [12, 14, 15]. The organophosphate, pirimiphos-methyl 50 EC was used in 2013, and pirimiphos-methyl 300 CS was used from 2014 to 2018 [14, 15]. As a result of the implementation of IRS in Atacora, there have been several insecticide resistance studies in the department that have used bioassays, biochemical tests, and molecular tests to characterize resistance levels in *Anopheles gambiae* s.l. populations [4, 7, 14, 16, 17]. These studies revealed suspected resistance, as well as resistance of mosquito vectors to bendiocarb and pyrethroids.

From 2017, IRS was gradually withdrawn from Atacora and transferred to other departments; In 2019, IRS was completely withdrawn, although one focal round of IRS did occur in 2020 in Kouandé commune [18]. However, only communes withdrawn in 2017 are involved in the present study. IRS withdrawal from Atacora was done to (1) reduce the pressure of insecticides from the multiple cycles of IRS, (2) assess if malaria transmission was adequately suppressed to allow for routine malaria services such as case management and insecticide treated bednets (ITNs) through continuous distribution to sustain malaria reduction, and (3) allow other districts to benefit from transmission reduction efficacy of IRS [14].

To determine if the withdrawal of IRS led to a decrease in resistance allele frequencies in populations of *An. gambiae* s.l., this study explores the changes in *kdr* (*L1014F*) and *ace*-1 (*G119S*) frequencies in *An. gambiae* s.l. populations at distinct benchmarks of IRS implementation: before IRS implementation (2010), during IRS implementation (2016), and 4 years after IRS withdrawal (2020).

## Methods

### Study area

**Fig. 1:**
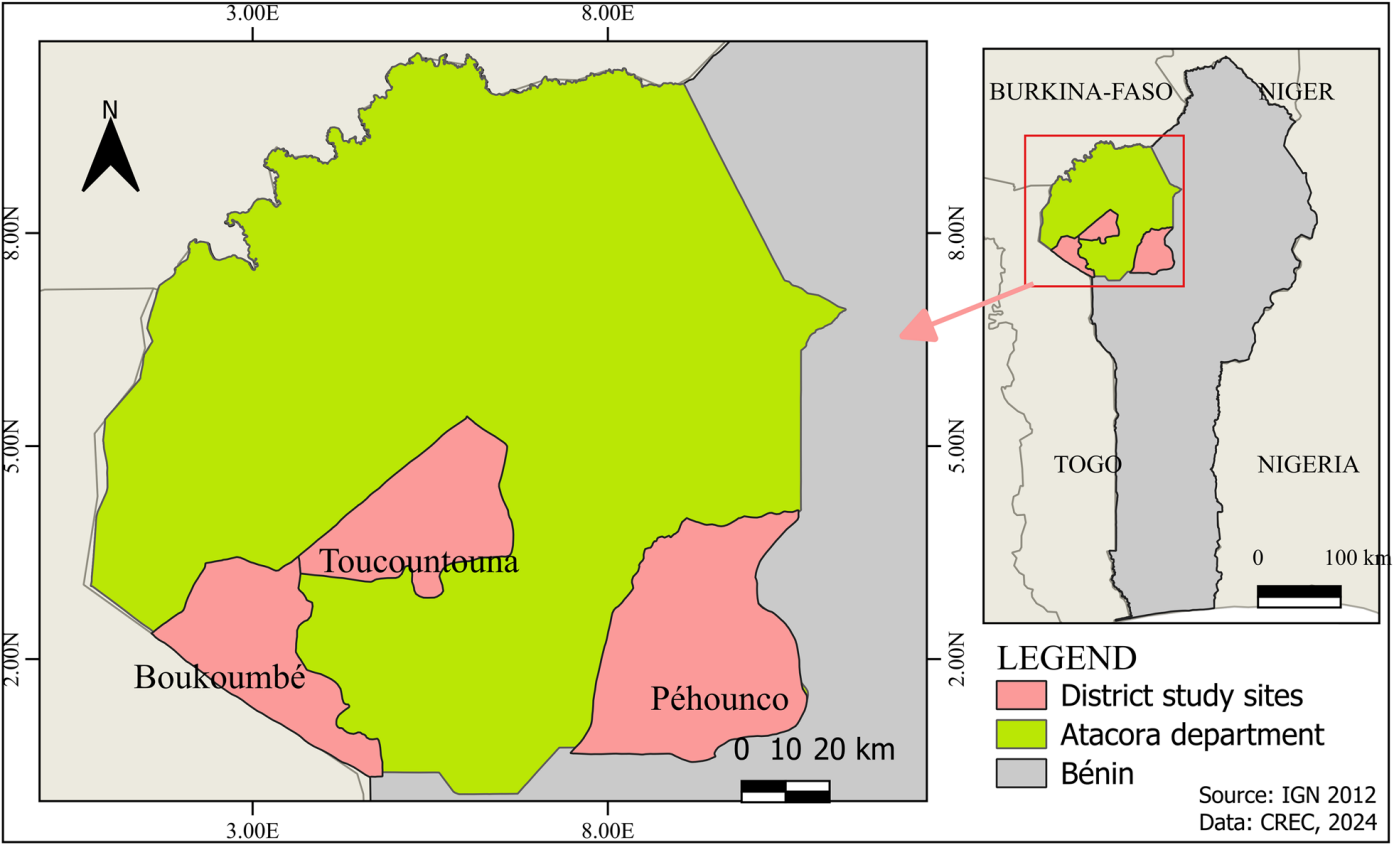
Map of the study area in the Atacora department of Benin

Figure 1 provides a map of the study sites in the Atacora department. The Atacora department has a total area of 20,499 km^2^ and a population of 772,262 people [19]. It is subdivided into nine districts, with Natitingou as the head town. The department of Atacora is a mountainous area with an average altitude of 700 m and the highest peak of 835 m is in Boukoumbé. It is the source of the major rivers of Benin and Togo [19]. The climate is tropical savanna with two seasons: the rainy season from June to October and the dry season from November to May. The soil is favorable to the cultivation of tubers and root crops (yam, cassava, and sweet potato), cereals (millet, maize, fonio, sorghum), and legumes [beans and voandzou (Bambara groundnut)], for which insecticides are used to control agricultural pests. The department’s predominant sectors of activity are “agriculture, fishing, and hunting” with a proportion of 77.2% [19]. Entomological and insecticide resistance monitoring of IRS has been conducted in the districts of Natitingou, Boukoumbé, Touncountouna, and Péhunco from 2010 to 2020. The data available in the district of Natitingou before and during the IRS were insufficient for comparison tests, so only the other three districts were included in this study**.**

### Mosquito collections

Adult mosquitoes collected before the IRS implementation period (2010) originated from pyrethrum spray catches. Mosquitoes collected during the IRS implementation period (2016) and 4 years after IRS withdrawal (2020) originated from human landing catches from the three study districts (Boukoumbé, Toucountouna, and Péhunco).

Mosquito samples before IRS had been collected during Benin’s rainy season (September/October). Samples used “during IRS implementation period” were collected in the dry season (March) and in the rainy season (June and August). Mosquito samples used 4 years after IRS withdrawal” were collected in the dry season (February/March) and in the wet season (September).

In each district, two villages were selected—a central village and a village on the periphery. Adult mosquitoes were captured during the two consecutive nights from 19.00 to 06.00 of each visit by local volunteers. Two houses were selected at random per villages. One collector inside and one collector outside were stationed at a household for hourly collections of mosquitoes at the level of each household. A total of 8 local catchers were selected per village and 16 per district. No insecticide-resistance bioassays were done on the collected mosquitoes as this activity was not initially considered at the time of the collection.

### Identification of sibling species in *Anopheles gambiae* complex

Collected mosquitoes were identified and separated at species level based on morphological criteria according to established taxonomic keys [20, 21]

Molecular characterization of *Anopheles gambiae* s.l. populations were performed on 1092 collected mosquitoes. The head and thorax of the mosquitoes were separated for further studies, while the rest of the body (abdomen, wings, and legs) was used for genomic DNA extraction and molecular characterization. DNA was extracted following the protocol of Myriam and Cécile [22].

### Identification of the different species

The extracted genomic DNA was used for molecular identification of the species of the *An. gambiae* s.l. complex. All mosquitoes were subjected to polymerase chain reaction (PCR) using the protocol of Scott et al. [23] to identify the different species of the *An. gambiae* complex.

The technique of Santolamazza et al. [24] was used to distinguish the twin species *An. gambiae* and *Anopheles coluzzii*. The PCR products are stored at a final temperature of 4 ℃ before being migrated by 1.5% agarose gel electrophoresis with ethidium bromide as an intercalating agent.

### Detection of the *L1014F* mutation on the *kdr* gene

The presence of the resistance allele (*L1014F*) of the *kdr* gene in samples collected from each study site was detected for each period by PCR following the protocol described by Martinez-Torres et al. [25].

The amplification program is composed of 40 cycles. Each cycle includes: initial denaturation at 94 ℃ for 1 min, hybridization at 48 ℃ for 2 min, and elongation at 72 ℃ for 2 min. Finally, this PCR ends with a final elongation at 72 ℃ for 10 min [23].

### Detection of the *G119S *mutation on the *ace-1* gene

The *G119S* mutation was detected for each period using mosquitoes collected according to the protocol of Weill et al. [26]. For this PCR, the following primers were used: Moustdir1 5′-CCGGGNGCSACYATGTGGAA-3′ and Moustrev1 5′-ACGATMACGTTCTCYTCCGA-3′, and the amplification program was as follows: 30 cycles and each cycle included denaturation at 94 ℃ for 30 s, hybridization at 52 ℃ for 30 s, and elongation at 72 ℃ for 1 min. The PCR products were digested with AluI restriction enzyme according to the manufacturer’s instructions before migration onto a 2% agarose gel.

### Statistical analysis

To calculate the allelic and genotypic frequencies of the *kdr* and *ace-1* genes in each district and by species, stratified 3 × 3 × 3 and 2 × 3 × 3 contingency tables were done in IBM-SPSS Statistics Subscription^®^ (Build 1.0.01406). Pearson’s chi-square (*χ*^2^) test was done for the comparison of proportions. Pairwise comparisons of column proportions were done using the *z*-test for proportions and the *P* values adjusted with the Bonferroni method to account for multiple comparisons. Frequencies of the mutant allele kdr L1014F and *ace-1*^R^ G119S were calculated using the formula:

$$F\left(R\right)=\frac{2nRR+nRS}{2(nRR+nRS+nSS)},$$

where F^®^ is the frequency of resistance, *n* is the number of mosquitoes of a given genotype, RR is the homozygous resistant genotype, RS is the heterozygous resistant genotype, and SS is the susceptible genotype [27]. A multivariable logistic regression was done in RStudio version 1.3.959 and R statistical software version 4.21 to determine the association (odds ratio) between the independent parameters: (1) IRS periods (before, during, and 4 years after IRS withdrawal), (2) location (Boukoumbé, Péhunco, and Toucountouna), and (3) mosquito species (*An. gambiae* s.s. versus non-*An. gambiae* s.s. sibling species); and the explanatory variables:

- the *kdr* logit—the homozygous resistant *kdr* genotype (1014F/1014F) versus the combined heterozygous resistant and homozygous susceptible *kdr* genotypes (1014L/1014F and 1014L/1014L), and
- the *ace-1* logit—the combined homozygous and heterozygous resistant genotypes (119S/119S and 119G/119S) versus the susceptible genotype (homozygous 119G).

### Genetic differentiation of the population

Hardy–Weinberg equilibrium was checked for each population with Genetics software version 4.7.5. The Weir and Cockerham [28] fixation index (*F*_*IS*_) was calculated using Genepop software.

All the mosquitoes from the Department of Atacora are considered here as the sample population. Thus, the different districts involved are considered as subpopulations, and the genetic differentiation of the population (*F*_*ST*_) was assessed before, during, and 4 years after the IRS.

The indices of genetic differentiation within populations (*F*_*ST*_) were calculated using the Genepop version 4.7.5 software and the criteria of Daniel Harlt [29] were used to assess them.

## Results

### Molecular identification of *Anopheles gambiae* s.l.

**Fig. 2:**
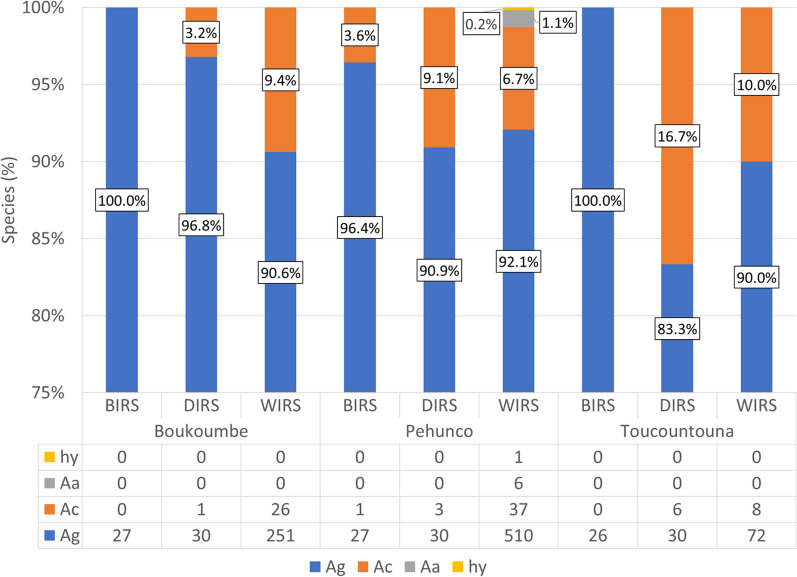
Percentage of *Anopheles gambiae* s.l. species collected by period and by district, before IRS (BIRS) in 2010, during IRS (DIRS) in 2016, and 4 years after IRS withdrawal (WIRS) in 2020; Ag, *An. gambiae* s.s.; Ac, *An. coluzzii*; Aa, *An. arabiensis*; hy, hybrids are *An. gambiae* s.s. and *An. coluzzii*

A total of 1092 mosquitoes were analyzed during the study. The different species obtained during the mosquito identification are displayed on the bar graph in Fig. 2. The bar graph presents the proportions and number of individuals of *An. gambiae* s.l. species collected in different districts and during different periods.

A Pearson’s chi-square analysis comparing *An. gambiae* s.s. and the other grouped *An. gambiae* s.l. sibling species by period and location showed that there was no statistically significant difference in the proportion of *An*. *gambiae* s.l. observed before, during, and 4 years after IRS (*χ*^2^ = 5.838, degree of freedom (*df*) = 2, *P* = 0.540), and there was no statistically significant difference in the proportion of mosquito species by location (*χ*^2^ = 0.656, *df* = 2, *P* = 0.720).

Before IRS, *An. gambiae* s.s. was nearly the exclusive *Anopheles* species collected in all districts; *An. coluzzii* was only found at 3.6% in Péhunco. During IRS, the percentage of *An. gambiae* s.s. ranged from 83.3% in Toucountouna to 96.8% in Boukoumbé, while the proportion of *An. coluzzii* was 3.2% in Boukoumbé, 9.1% in Péhunco, and 16.7% in Toucountouna. Four years after IRS, *An. gambiae* s.s. remained the dominant *Anopheles* species in all districts with proportions of 90.6% in Boukoumbé, 92.1% in Péhunco, and 90.0% in Toucountouna; the percentage of *An. coluzzii* was 9.4% in Boukoumbé, 6.7% in Péhunco, and 10.0% in Toucountouna. *An. arabiensis* and a hybrid form of *An. gambiae* s.s. and *An. coluzzii* were also found in Péhunco during this period, albeit at very low percentages (1.1% and 0.2%, respectively).

### Prevalence of *kdr* mutations

**Fig. 3:**
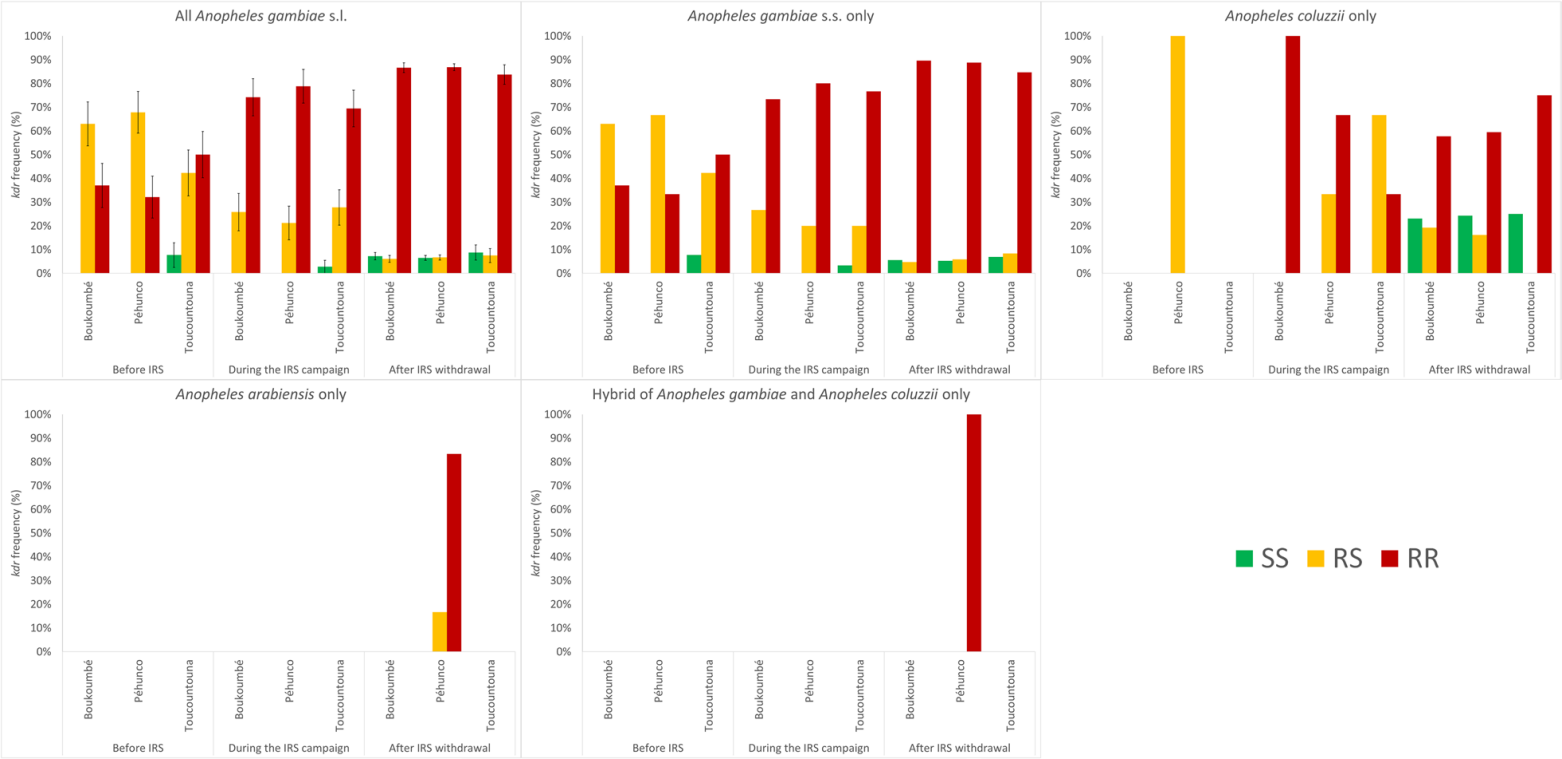
Bar chart showing the *kdr* allelle frequency (percentage with standard error bars in the first panel) in *An. gambiae* s.l. in the districts of the Atacora department of Benin by species and period

Tables 1 and 2 and Fig. 3 illustrate frequencies of the *kdr* alleles at different periods (before IRS, during IRS, and 4 years after IRS withdrawal) stratified by location (Boukoumbé, Péhunco, and Toucountouna districts) and species (*An. gambiae*s.s. only and other grouped *An. gambiae* s.l. sibling species). Before IRS, during IRS and 4 years after IRS withdrawal, the frequencies of the *kdr* gene for *An*. *gambiae* s.l. populations were 69%, 87%, and 90%, respectively, for the district of Boukoumbé; 66%, 89%, and 90% for the district of Péhunco; and 71%, 83%, and 88% for district the of Toucountouna. During the same periods, *kdr* gene frequencies for *An*. *gambiae* s.s. only populations were 69%, 88%, and 92% for all districts. The frequencies for non-*An*. *gambiae* s.s. were 50%, 75%, and 71% in the same districts. The period was significantly associated with the frequency of the *kdr* genotypes irrespective of location (Table 1) and species (Table 2) stratification. Tables 1 and 2 and Fig. 3 show that the *1014F kdr* allele frequency significantly increased over time, where the aggregated frequency was 69% [95% confidence interval (CI) 61–76%] before IRS, 87% (95% CI 82–91%) during IRS, and 90% (95% CI 88–91%) 4 years after IRS withdrawal.

**Table 1:** Percentages of the genotypic (*n*) and the mutant [*1014F*] allelic (95% confidence interval) frequencies of the *kdr* gene in *Anopheles gambiae* s.l. populations by period and location using a layered 3 × 3 × 3 contingency table

Location	Period	Mos (*n*)	RR 1014F/1014F	RS 1014L/1014F	SS 1014L/1014L	*F*^†^ [1014F allele]	*P*
Boukoumbé	BIRS	27	37% (10)^a^	63% (17)^a^	0% (0)	69% (56–81%)	< 0.001
	DIRS	31	74% (23)^b^	26% (8)^b^	0% (0)	87% (79–95%)	
	WIRS	277	87% (240)^b^	6% (17)^c^	7% (20)	90% (87–92%)	
Péhunco	BIRS	28	32% (9)^a^	68% (19)^a^	0% (0)	66% (54–78%)	< 0.001
	DIRS	33	79% (26)^b^	21% (7)^b^	0% (0)	89% (82–97%)	
	WIRS	554	87% (481)^b^	7% (37)^c^	7% (36)	90% (88–92%)	
Toucountouna	BIRS	26	50% (13)^a^	42% (11)^a^	8% (2)^a^	71% (59–83%)	0.001
	DIRS	36	69% (25)^a,b^	28% (10)^a^	3% (1)^a^	83% (75–92%)	
	WIRS	80	84% (67)^b^	8% (6)^b^	9% (7)^a^	88% (82–93%)	
Total	BIRS	81	40% (32)^a^	58% (47)^a^	3% (2)^a^	69% (61–76%)	< 0.001
	DIRS	100	74% (74)^b^	25% (25)^b^	1% (1)^a^	87% (82–91%)	
	WIRS	911	87% (788)^c^	7% (60)^c^	7% (63)^a^	90% (88–91%)	

Each superscript letter denotes a subset of genotype categories whose column proportions do not differ significantly from each other at the 0.05 level. BIRS, before IRS; DIRS (2010), during IRS (2016); WIRS, 4 years after IRS withdrawal (2020). ^†^Frequency of the *1014F* allele with the 95% confidence interval

**Table 2:** Percentages of the genotypic (*n*) and the mutant [*1014F*] allelic (95% confidence interval) frequencies of the *kdr* gene in *Anopheles gambiae* s.l. populations by period and species using a layered 2 × 3 × 3 contingency table

Species	Period	Mos (*n*)	RR 1014F/1014F	RS 1014L/1014F	SS 1014L/1014L	*F*^†^ [1014F allele]	*P*
Ag	BIRS	80	40% (32)^a^	58% (46)^a^	3% (2)^a^	69% (62–76%)	< 0.001
	DIRS	90	77% (69)^b^	22% (20)^b^	1% (1)^a^	88% (83–93%)	
	WIRS	833	89% (739)^c^	6% (48)^c^	6% (46)^a^	92% (90–93%)	
Non-Ag	BIRS	1	0% (0)	100% (1)^a,b^	0% (0)	50% (0–0%)	0.020
	DIRS	10	50% (5)^a^	50% (5)^b^	0% (0)	75% (56–94%)	
	WIRS	78	63% (49)^a^	15% (12)^a^	22% (17)	71% (63–78%)	
Total	BIRS	81	40% (32)^a^	58% (47)^a^	3% (2)^a^	69% (61–76%)	< 0.001
	DIRS	100	74% (74)^b^	25% (25)^b^	1% (1)^a^	87% (82–91%)	
	WIRS	911	87% (788)^c^	7% (60)^c^	7% (63)^a^	90% (88–91%)	

Each superscript letter denotes a subset of genotype categories whose column proportions do not differ significantly from each other at the 0.05 level. BIRS, before IRS; DIRS (2010), during IRS (2016); WIRS, 4 years after IRS withdrawal (2020); Ag, *An. gambaiae* s.s. only; Non-Ag, all other *An. gambiae* s.l. sibling species. ^†^Frequency of the *1014F* allele with the 95% confidence interval

While location and *kdr* genotypes were not significantly associated (*χ*^2^ = 9.101; *df* = 4; *P* = 0.059), mosquito species were significantly associated with *kdr* genotypes (*χ*^2^ = 37.957; *df* = 2; *P* < 0.001). This association seems to be driven by the period 4 years after IRS withdrawal, where *An. gambiae* s.s. only had high frequency of homozygous (1014F/1014F) *kdr* mutant genotypes (RR of 89%, RS of 6%, and SS of 6%) and non-*An. gambiae* s.s had a lower frequency of homozygous *kdr*-mutant genotypes (RR of 63%, RS of 15%, and SS of 22%).

Odds ratios of a multivariable logistic regression (Table 5) showed a significant increase an ~5 and ~12 times the odds of mosquitoes being homozygous for the *1014F* allele when comparing the periods, during IRS, and 4 years after IRS withdrawal with the before IRS period, respectively. There was no difference in the odds of mosquitoes being homozygous for *1014F* by location. Also, non-*An. gambiae* s.s. had a significant ~80% decrease in the odds of being homozygous for *1014F* compared with *An. gambiae* s.s.

### Prevalence of ace-1 mutations

**Fig. 4:**
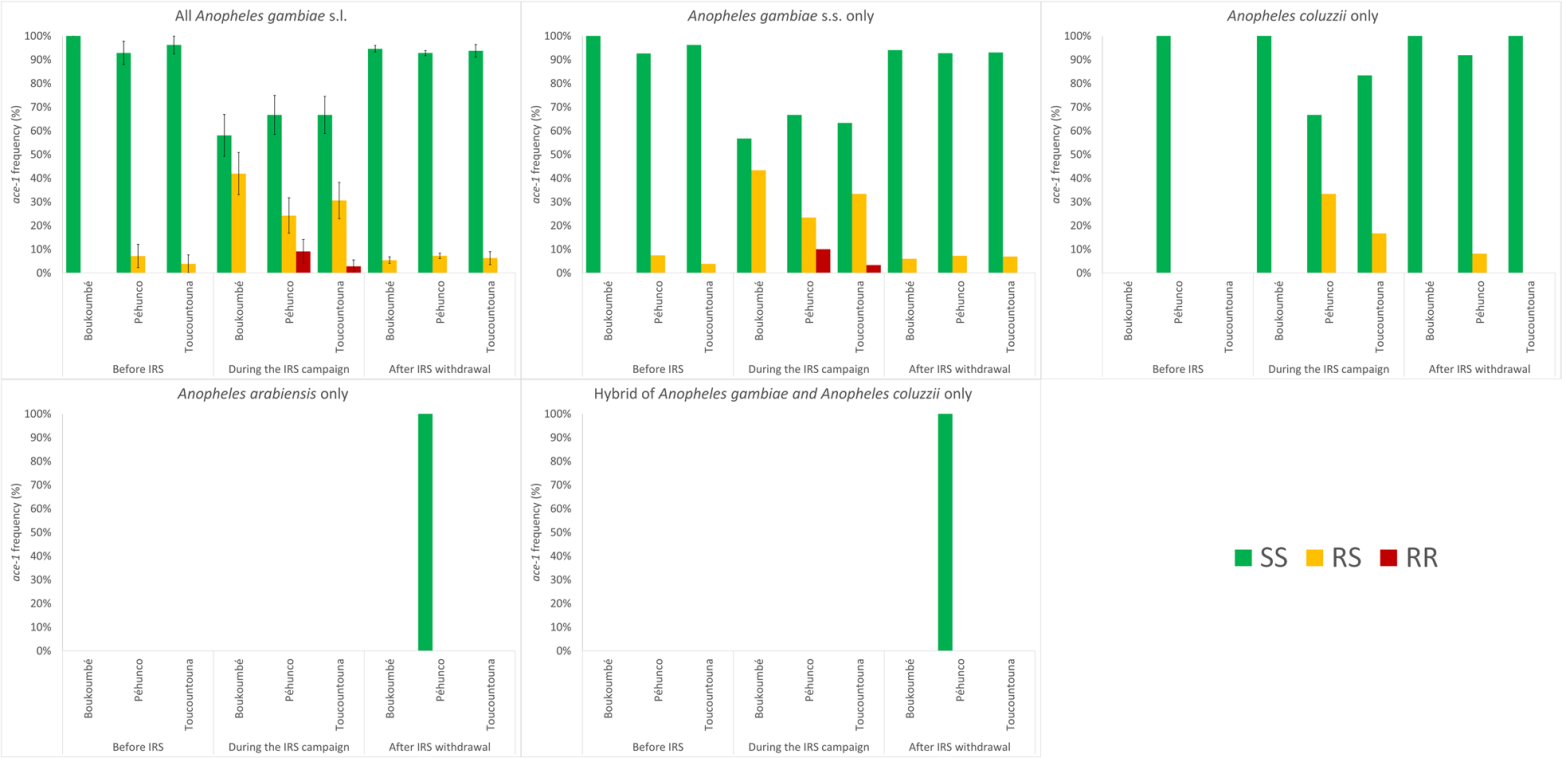
Bar chart showing the evolution *ace-1* allele frequency (percentage with standard error bars in the first panel) in *An. gambiae* s.l. in the districts of the Atacora department of Benin by species and period

Tables 3 and 4 and Fig. 4 show frequencies of the *ace-1* alleles at different periods (before IRS, during IRS, and 4 years after IRS withdrawal) stratified by location (Boukoumbé, Péhunco, and Toucountouna districts), and species (*An. gambiae* s.s. only and other grouped *An. gambiae* s.l. sibling species). Before IRS, during IRS, and 4 years after IRS withdrawal, the frequencies of the *ace-1* gene for *An*. *gambiae* s.l. populations were 0%, 21%, and 3%, respectively, for the district of Boukoumbé; 4%, 21%, and 4% for district of Péhunco; and 2%, 18%, and 3% for district of Toucountouna. These frequencies were 2%, 21%, and 3% for *An*. *gambiae* s.s. only before IRS, during IRS and 4 years after IRS withdrawal versus 0%, 10%, and 2% for non-*An*. *gambiae* s.s., in all the study districts.

**Table 3:** Percentages of the genotypic (*n*) and the mutant [*119S*] allelic (95% confidence interval) frequencies of the *ace-1* gene in *Anopheles gambiae* s.l. populations by period and location using a layered 3 × 3 × 3 contingency table

Location	Period	Mos (*n*)	RR 119S/119S	RS 119G/119S	SS 119G/119G	*F*^†^ [119S allele]	*P*
Boukoumbé	BIRS	27	0% (0)	0% (0)	100% (27)^a^	0% (0–0%)	< 0.001
	DIRS	31	0% (0)	42% (13)^b^	58% (18)^b^	21% (11–31%)	
	WIRS	277	0% (0)	5% (15)^a^	95% (262)^a^	3% (1–4%)	
Péhunco	BIRS	28	0% (0)	7% (2)^a,b^	93% (26)^a^	4% (−1% to 8%)	< 0.001
	DIRS	33	9% (3)	24% (8)^b^	67% (22)^b^	21% (11–31%)	
	WIRS	554	0% (0)	7% (40)^a^	93% (514)^a^	4% (3–5%)	
Toucountouna	BIRS	26	0% (0)	4% (1)^a^	96% (25)^a^	2% (−2% to 6%)	0.001
	DIRS	36	3% (1)	31% (11)^b^	67% (24)^b^	18% (9–27%)	
	WIRS	80	0% (0)	6% (5)^a^	94% (75)^a^	3% (0–6%)	
Total	BIRS	81	0% (0)	4% (3)^a^	96% (78)^a^	2% (0–4%)	< 0.001
	DIRS	100	4% (4)	32% (32)^b^	64% (64)^b^	20% (14–26%)	
	WIRS	911	0% (0)	7% (60)^a^	93% (851)^a^	3% (2–4%)	

Each superscript letter denotes a subset of genotype categories whose column proportions do not differ significantly from each other at the 0.05 level. BIRS, before IRS; DIRS (2010), during IRS (2016); WIRS, 4 years after IRS withdrawal (2020). ^†^Frequency of the *119S* allele with the 95% confidence interval

**Table 4:** Percentages of the genotypic (*n*) and the mutant [*119S*] allelic (95% confidence interval) frequencies of the *ace-1* gene in *Anopheles gambiae* s.l. populations by period and species using a layered 2 × 3 × 3 contingency table

Location	Period	Mos (*n*)	RR 119S/119S	RS 119G/119S	SS 119G/119G	*F*^†^ [119S allele]	*P*
Ag	BIRS	80	0% (0)	4% (3)^a^	96% (77)^a^	2% (0–4%)	< 0.001
	DIRS	90	4% (4)	33% (30)^b^	62% (56)^b^	21% (15–27%)	
	WIRS	833	0% (0)	7% (57)^a^	93% (776)^a^	3% (3–4%)	
Non-Ag	BIRS	1	0% (0)	0% (0)	100% (1)^a^	0% (0–0%)	
	DIRS	10	0% (0)	20% (2)^a^	80% (8)^a^	10% (−3% to –23%)	0.110
	WIRS	78	0% (0)	4% (3)^a^	96% (75)^a^	2% (0–4%)	
Total	BIRS	81	0% (0)	4% (3)^a^	96% (78)^a^	2% (0–4%)	< 0.001
	DIRS	100	4% (4)	32% (32)^b^	64% (64)^b^	20% (14–26%)	
	WIRS	911	0% (0)	7% (60)^a^	93% (851)^a^	3% (2–4%)	

Each superscript letter denotes a subset of genotype categories whose column proportions do not differ significantly from each other at the 0.05 level. BIRS, before IRS; DIRS (2010), during IRS (2016); WIRS, 4 years after IRS withdrawal (2020); Ag, *An. gambiae* s.s. only; Non-Ag: All other *An. gambiae* s.l. sibling species. ^†^Frequency of the 119S allele with the 95% confidence interval

The period was significantly associated with the frequency of the *ace-1*genotypes irrespective of the location (Table 3) stratification. However, the association between period and the *ace-1* genotypes varied by the species stratification; Table 4 presents that in the *An. gambiae* s.s. only strata the association was statistically significant (*χ*^2^ = 115.623; *df* = 4; *P* < 0.001) and in the non-*An. gambiae* s.s. strata the association was not statistically significant (*χ*^2^ = 4.422; *df* = 2; *P* = 0.110).

Tables 3 and 4 and Fig. 4 show that the 119S *ace-1* allele frequency significantly increased during IRS but was low before and after IRS; the aggregated frequency was 2% (95% CI 0–4%) before IRS, 20% (95% CI 14–26%) during IRS, and 3% (95% CI 2–4%) 4 years after IRS withdrawal.

Neither location (*χ*^2^ = 4.175; *df* = 4; *P* = 0.383) or species (*χ*^2^ = 1.59; *df* = 2; *P* = 0.463) was significantly associated with *ace*-1 genotypes.

For odds ratios from multivariate logistic regression (Table 5) for *ace-1*, there were an ~16× (significant) and ~2× (not significant) increase in the odds of mosquitoes carrying a 116S allele when comparing the periods, during IRS, and 4 years after IRS withdrawal with the before IRS period, respectively. There was no significant difference in the odds of mosquitoes carrying the *116S* allele by location. There was also no significant difference in the odds of non-*An. gambiae* s.s. and *An. gambiae* s.s. carrying the *116S* allele.

**Table 5:** Results of a multivariable logistic regression models indicating the association (odds ratio) between the independent variables of IRS-period, location, and species; dependent variables of the homozygous mutant genotypes of *kdr* (*1014F/1014F*); and the combined homozygous and heterozygous mutant genotypes of *ace-1* (*119S/119S* and *119G/119S*)

Parameters	Variables	Odd ratio (95% confidence interval)	
		kdr^*†*^	*ace-1*^*‡*^
	(Intercept)	0.66 (0.39–1.09)	0.04 (0.01–0.10)
Period	During IRS (2010)	5.17 (2.75–9.99)	15.54 (5.27–66.65)
	Withdrawal of IRS (2016)	11.75 (7.11–19.72)	1.83 (0.65–7.66)
	Before IRS (2020)	1*	1*
Location	Péhunco	1.02 (0.70–1.48)	1.21 (0.74–2.04)
	Toucountouna	1.01 (0.60–1.72)	1.04 (0.51–2.05)
	Boukoumbé	1*	1*
Sibling species	Non-*An. gambiae* s.s	0.22 (0.14–0.36)	0.49 (0.16–1.18)
	*An. gambiae* s.s. only	1*	1*

^†^The dependent variable was the logit of the homozygous mutant *kdr* genotype (1014F/1014F) and the heterozygous mutant and homozygous susceptible *kdr* genotypes (*1014L/1014F* and *1014L/1014L*). ^‡^The dependent variable was the logit of the combined homozygous and heterozygous mutant genotypes (*119S/119S* and *119G/119S*) and the susceptible genotype (homozygous *119G*). ^*^Reference group

### Hardy–Weinberg equilibrium

Some populations did not follow the Hardy–Weinberg equilibrium and for this reason, the fixation index (*F*_*IS*_) was calculated to assess the evolutionary forces in place. Table 6 shows the fixation indices of the two alleles *L1014F* and *G119S.*

**Table 6:** Variation in observed and expected heterozygosity and fixation index

Period	Districts	Number tested	*L 1014F* mutation of *kdr* gene			*G 119S* mutation of a*ce-1* gene		
			*H*_*o*_	*H*_*e*_	*F*_IS_ *(W&C)*	*Ho*	*He*	*F*_IS_* (W&C)*
BIRS	Boukoumbé	27	0.630	0.440	−0.4444	0.000	0.000	NA
	Péhunco	27	0.667	0.453	− 0.4857	0.074	0.073	− 0.0196
	Toucountouna	26	0.423	0.419	− 0.0110	0.038	0.038	− 0.0000
DIRS	Boukoumbé	30	0.267	0.235	− 0.1373	0.433	0.345	− 0.2609
	Péhunco	30	0.200	0.183	− 0.0943	0.233	0.345	0.3278
	Toucountouna	30	0.200	0.235	− 0.1512	0.333	0.325	− 0.0247
WIRS	Boukoumbé	251	0.048	0.147	0.6751	0.060	0.058	− 0.0288
	Péhunco	510	0.059	0.151	0.6114	0.073	0.070	− 0.0367
	Toucountouna	72	0.083	0.199	0.5828	0.069	0.081	− 0.0294

BIRS, before IRS; DIRS (2010), during IRS (2016); WIRS, 4 years after IRS withdrawal; (2020); *H*_*o*_, observed heterozygosity; *H*_*e*_, expected heterozygosity; *F*_*IS*_, fixation index

Analysis of this table revealed that for the *L1014F* allele, the fixation index is negative showing an excess of heterozygosity (*F*_*IS*_ < 0) before and during IRS, while 4 years after IRS withdrawal a deficit of heterozygosity (*F*_*IS*_ > 0) was observed in all periods. On the other hand, concerning the *ace-1* gene, except for the Péhunco population, where a heterozygosity deficit was observed during IRS due to either consanguinity (mating between two related individuals with at least one verifiable common ancestor) of the reproductive regime within the population or genetic drift (the process of changing the frequency of an allele in a population over time). Excess heterozygosity, which may be due to overall super dominance spread over many loci in the genome or to the presence of many recessive deleterious alleles, has been found in other populations over various periods.

### Genetic differentiation within different populations of *Anopheles gambiae*

Since *An. gambiae* s.l. populations from different periods were not subjected to the same pressures, and genetic differentiation within populations was calculated to better assess the impact of these insecticides. Table 7 presents the genetic differentiation (*F*_*ST*_) calculated in the populations.

**Table 7:** Genetic differentiation within populations before, during and after IRS withdrawal

Period	*F*_ST_ (W&C) *L1014F*	*F*_ST_ (W&C) *G119S*
Before IRS (2010)	−0.010	0.000
During IRS (2016)	−0.016	−0.013
Four years after IRS withdrawal (2020)	−0.001	−0.001

From the analysis of this table, it appears that at the level of the two genes, no genetic differentiation (*F*_*ST*_ ≤ 0) was observed during the different periods in the populations.

## Discussion

This study provides initial insights into how changes in insecticide pressure from IRS implementation and withdrawal may have altered the genotypic profile of *An. gambiae* s.l. in Atacora in comparison with before IRS implementation. The two insecticide resistance markers investigated were the target site mutations in the knockdown resistance (*kdr*) gene (*L1014F*) and the acetylcholinesterase (*ace-1*) resistance gene (*G119S*); these markers are commonly studied genes and were considered good candidates to understand how the change in IRS insecticide pressure affects the genetic profile of malaria vectors in Atacora [3, 30].

Because vector behavior and resistance levels can vary widely, it is crucial to compare vector densities and resistance levels. The three species of *Anopheles gambiae* s.l. complex found in the study area were *An. gambiae* s.s., *An. coluzzii*, and *An. arabiensis*, where *An. gambiae* s.s. was the most abundant vector collected; this suggests that resistance levels in this vector likely determined the impact of insecticide-based vector control for Atacora. The low numbers of *An. coluzzii* and *An. arabiensis* are thought to be due to the ecology of these species in Benin, which prefers permanent, mostly artificial larval sites, while semipermanent and temporary habitats are more favorable to *An. gambiae* s.s. [31]. The disparity observed between *An. coluzzii* populations before and during the IRS could be attributed to the sampling period. In fact, data collected before the launch of the IRS were limited to the rainy season, whereas data collected during the IRS covered both the dry and rainy seasons.

The frequencies of the *kdr* alleles in the *Anopheles gambiae* s.l. populations increased in the direction of homozygous resistance (*L1014F/ L1014F*) with an increased frequency of the *L1014F* mutation in individuals over time (before IRS, during IRS and, 4 years after IRS withdrawal). Before the introduction of IRS in Atacora, standard LLINs were distributed, and ITN implementation continued throughout the study timeframe and beyond. A compilation of ITN indicators using data from Demographic and Health Survey (DHS) surveys and Malaria Indicator Cluster Surveys (MICS) from 2006 to 2018 showed that ITN ownership ranged from 26% to 94%, ITN access ranged from 14% to 82%, ITN use ranged from 13 to 68%; the 2006 DHS survey serves as the baseline and was the lowest for all indicators [32]. Because mutations in the *kdr* gene are usually associated with pyrethroid insecticide pressure, one likely reason for the continued increase in resistant *kdr* alleles before IRS implementation and beyond is due to ITN distribution. ITN distribution continues in Atacora with PBO and/or pyrethroid-chlorfenapyr ITNs being planned for future distribution in 2023. It may be useful to determine if these ITNs will continue to impact *kdr* allele frequency in Atacora.

Several studies have shown that the mode of action for carbamates and organophosphates is to inhibit *ace-1* [33, 34]. The increase in the frequency of the *G119S* allele on the *ace-1* gene observed during IRS implementation in the different districts showed that the carbamates and organophosphates did exert pressure on the mosquito population toward resistance. However, 4 years after IRS withdrawal, the mosquito population seemed to have reverted to pre-IRS *ace-1* allele frequency levels. This result generally favors the notion that reducing the pressure of insecticides from the environment would cause mosquitoes to regain susceptibility to an insecticide; hence, the rotation of insecticide classes may be a key strategy for the management of resistance.

This study had some limitations. While *kdr* and *ace-1* are common insecticide resistance markers included in resistance studies, bioassay results were not available in this study to determine the mortality rates of mosquitoes after exposure to pyrethroids, carbamates, and organophosphates at different periods. The availability of bioassay data would have been useful, as some studies suggested that there is not a perfect association between the mortality rates of mosquitoes in bioassay and molecular markers [7, 17]. However, target site mutations in key genes may still serve as useful markers for estimating the selective pressure of insecticides on mosquito genetics in the absence of phenotype data. In the study, mosquitoes were also sourced from PSCs and human landing catches rather than a larval mosquito leading to an un-uniform age structure of mosquitoes being assessed. This limitation is important for bioassays but may be less important for target site mutation studies, as it was assumed that these mutations would not drastically change during the life history of individual mosquitoes, whereas mosquito age may affect bioassay results [35]. In addition, the season when the mosquitoes were collected also may have affected the results, though, apart from before IRS implementation, both rainy season and dry season mosquito samples were included in the analysis. In this study, we did not have a comparison of non-IRS sites to be sure that the association between IRS implementation and withdrawal was not spurious and confounded by a coincidental factor such as a change in agriculture pesticide use. Furthermore, in this study, we did not have agricultural insecticide-use data, which may play some role in shaping the selective pressure and genotypic profile of the mosquito populations [36–38]. The lack of this information suggests that caution should be taken when interpreting the results. There was a low and variable sample size before and during IRS implementation, as well as 4 years after IRS withdrawal. In 2010 and 2016, the objectives pursued by vector control programs were more on transmission, so few mosquitoes were analyzed by PCR. While the limited availability of mosquito samples prevented greater confidence in the results, the findings do seem to follow a logical trend, and statistical significance was found in most of the analyses. A final limitation is that this study solely focused on *kdr* and *ace-1*; the association of IRS with other insecticide resistance mechanisms (such as oxidase- or cuticular-protein-mediated resistance) may lead to a more insightful understanding of the impact of IRS implementation and withdrawal. Nevertheless, these results with *kdr* and *ace-1* in Atacora still contribute to the knowledge base of insecticide resistance.

Other studies have looked at the change in insecticide resistance markers before and after vector control intervention and have shown that there are changes in molecular profiles that occur, which are associated with the intervention implementation [3, 30]. This study aligns with those findings.

## Conclusions

The frequency of *kdr (L1014F)* and *ace-1* (*G119S)* mutations in *Anopheles gambiae* s.l. 4 years after the withdrawal of IRS from the Atacora department found that the frequencies of the *L1014F* mutation continued increasing in the population even after the withdrawal of IRS, while the frequency of the *G119S* allele increased during IRS and decreased 4 years after the withdrawal of IRS in all populations. This study provides evidence of how insecticide pressure influences mosquito genotypes at key loci. While Benin is currently not conducting IRS, this study highlights that to preserve susceptibility to the insecticides used for IRS; it may be preferable to withdraw the intervention after several years of implementation or to alternate with the use of other classes of insecticides targeting different resistance mechanisms.

## Data Availability

The data supporting the fndings of the study must be available within the article and/or its supplementary materials, or deposited in a publicly available database.

## Abbreviations

*ace*-1: Acetylcholinesterase-1
CDC: US Centers for Disease Control and Prevention
CREC: Centre de Recherche Entomologique de Cotonou
IRS: Indoor residual spraying
ITNs: Insecticide-treated bed nets
LLINs: Long-lasting insecticidal nets
*kdr*: Knockdown resistance
NMCP: National Malaria Control Programme
PCR: Polymerase chain reaction
PMI: US President’s Malaria Initiative
s.l.: Sensu lato
s.s.: Sensu stricto
USAID: United States Agency for International Development
WHO: World Health Organization
